# Nutritional Deficiencies, the Absence of Information and Caregiver Shortcomings: A Qualitative Analysis of Infant Feeding Practices in Rural China

**DOI:** 10.1371/journal.pone.0153385

**Published:** 2016-04-13

**Authors:** Ai Yue, Lauren Marsh, Huan Zhou, Alexis Medina, Renfu Luo, Yaojiang Shi, Linxiu Zhang, Kaleigh Kenny, Scott Rozelle

**Affiliations:** 1 Center for Experimental Economics in Education (CEEE), Shaanxi Normal University, Xi’an, Shaanxi, 710119, China; 2 West China School of Public Health, Sichuan University, Chengdu, Sichuan, 610041, China; 3 Rural Education Action Program, Freeman Spogli Institute for International Studies, Stanford University, Stanford, California, 94305, United States of America; 4 School of Advanced Agricultural Sciences, Peking University, Beijing, 100871, China; TNO, NETHERLANDS

## Abstract

**Background and Objectives:**

Development during the first two years of life is critical and has a lasting impact on a child’s health. Poor infant and child nutrition can lead to deficiencies in essential micronutrients, which may cause a weakened immune system and lasting effects on children's growth and development. Recent studies in rural Shaanxi Province found an anemia prevalence of 54.3% among rural children aged six to twelve months. While new large-scale, quantitative research has begun to catalogue the extent of child malnutrition and anemia, no effort has yet been made to look more closely at the potential reasons for rural children’s nutritional deficiencies through qualitative analysis. This study aims to elucidate some of the fundamental causes of poor complementary feeding practices that may lead to anemia among children in rural Shaanxi Province, China.

**Methodology:**

We interviewed sixty caregivers participating in a large survey on child health and nutrition. We conducted three waves of interviews with children’s primary caregivers in seventeen rural villages within four nationally-designated poverty counties in the southern part of Shaanxi Province.

**Results:**

The qualitative analysis reveals that poor complementary feeding practices are common across our sample. Information gathered from our interviews suggests that complementary feeding practices are impeded by two constraints: absence of understanding topics related to infant health and nutrition under caregivers, as well as inadequate sources of information on these topics. Poverty does not appear to constrain child feeding practices.

**Conclusion:**

Our results uncover lack of proper knowledge on infant and child nutrition among rural caregivers in China. This situation causes them to fail incorporating micronutrient rich foods in their children's diet. Age-appropriate complementary feeding can stimulate children’s physical and cognitive development, but in its absence it leads to iron-deficiency anemia. We suggest that steps be taken to educate caregivers to improve complementary feeding of their infants and children.

## Introduction

In recent decades, China has made vast improvements in child health and nutrition. Between 1990 and 2013 the share of children under the age of 5 years-old that were malnourished (as measured by the proportion of the population that was underweight) decreased from 13.37% to 1.37% and infant mortality rates decreased from 50.26% to 9.5% [1]. These improvements were brought about, in part, due to governmental efforts aimed at decreasing poverty and improving the overall health status of China’s population. Policy makers demonstrated an increased commitment to overall health improvements as they formulated China’s 12^th^ five-year plan for national development in the mid-2000s. In addition to laying out plans for economic development, China introduced plans for improvements to the health sector, in terms of both health care allocation and improvements in individual health status [2].

Despite these vast improvements, gaps in development and overall health still exist between urban and rural areas. Although studies have demonstrated that this gap is diminishing [3], findings suggest that those living in rural areas remain at a disadvantage compared to those residing in urban centers [4]. The central government has demonstrated increased interest in this issue in recent years [2] and has sought ways to improve rural health and nutrition through the implementation of new targeted programs [5]. While these initiatives may have led to some improvements in the health status of rural populations, they appear to be missing one of its most vulnerable demographic groups: young children.

Proper health and nutrition practices are integral to early childhood growth and development. Poor nutrition can lead to micronutrient deficiencies which may cause weakening of the immune system and have lasting effects on children's growth and development, human capital accumulation, and lifetime earnings [6, 7]. Given that today’s rural children represent a large portion of China’s future labor force, ensuring that these children are adequately nourished is critical to the nation’s development. However, there is growing evidence that young children in China’s rural areas still often suffer from malnutrition, in terms of both undernutrition and nutrient imbalances. In 2008, it was found that in rural areas of China 34% of children under six months-of-age had anemia, and 21% of children under two years-of-age were stunted [8]. Recent research also suggests that malnutrition arising from micronutrient imbalances is a particularly serious issue in rural China. A recent study conducted in rural Shaanxi Province found that anemia was common among infants six to eleven months of age, with a prevalence of 54.3%. However, this same study revealed that stunting was uncommon as it was only exhibited by 1.2% to 3.7% of the study population [9, 10]. Although children could develop anemia for a number of reasons, it has been postulated from a nearly-nationally representative study that 90% of the anemia in children under six years-of-age in China results from iron-deficiency [11]. Despite the fact that these two samples varied slightly in age, this evidence suggests that one of the main problems facing poor rural children in China is malnutrition arising from micronutrient-deficient diets.

To a large extent, these poor nutritional outcomes may be associated with insufficient diets due to inadequate complementary feeding practices. Complementary feeding encompasses weaning, when infants progress from exclusive breast-milk or formula diets to eating solid foods. This process typically occurs between the ages of 6–24 months, a time that has been highlighted as a critical developmental period in which micronutrient deficiencies lead to undernutrition of a large portion of the world’s children [12]. The World Health Organization (WHO) recommends that children begin to be introduced to complementary foods starting at six months of age, that children eat some form of meat, poultry, fish or eggs, as well as some form of fruit or vegetable daily. The WHO also stresses that at this stage a vegetarian diet is insufficient to support the micronutrient levels necessary for adequate child development and encourages caregivers to provide children with supplements or vitamin-fortified food [13].

Previous research on the complementary feeding practices of caregivers of young children in rural China demonstrate that children’s diets lack variety and seldom include meat. Quantitative data collected by Luo et al. (2014) revealed that only 23.7 percent of the 6–11 month-old children included in the study had consumed meat and only 54.6 percent had consumed vegetables in the past week [9]. These findings are of particular concern as recent research has demonstrated that the period before a child reaches 18 months of age may be critical for the prevention and treatment of iron-deficiency anemia [14], suggesting that the effects of poor feeding practices at this stage in a child’s life may affect their development and health status over the long-run. The Chinese government has recognized its commitment to reducing the prevalence of childhood malnutrition and anemia and encouraging scientific feeding of infants and young children [2].

Given the high prevalence of poor nutritional outcomes that can be alleviated and avoided by proper complementary feeding, this study attempts to identify the root causes of poor-quality infant nutrition in rural Shaanxi Province, China. Within this research, we seek to make use of a mixed-methods research approach to investigate the underlying factors prevalent in rural communities that contribute to poor complementary feeding practices in the hopes of informing future policy solutions that can be designed to mitigate their effects. While new large-scale, quantitative research has started to catalogue the extent of child malnutrition in rural China, less effort has been made to use a qualitative approach to look more closely at the potential reasons for rural children’s nutritional deficiencies.

Our objectives are (1) to understand current complementary feeding practices in order to identify areas in which these practices are deficient; and (2) to identify the factors that impede caregivers from practicing proper complementary feeding. We anticipate that the findings will be valuable for informing the design and implementation of effective nutritional education campaigns in China as well as broader public health efforts.

## Methods

### Ethical Approval

This study received ethical approval from the Stanford University Institutional Review Board (IRB) (Protocol ID 25734) and from the Sichuan University Ethical Review Board (Protocol ID 2013005–01). The Chinese National Health and Family Planning Commission granted permission for the field work.

### Sample Selection

Our sample is comprised of three waves of qualitative interviews conducted between July 2013 and May 2015. All three sets of interviews were conducted with children’s primary caregivers in seventeen rural villages within four nationally-designated poverty counties in the southern part of Shaanxi Province that have been determined to have high rates of child anemia [9, 10]. Further details on the three waves of interviews are provided in Table 1.

**Table 1:** Sample Selection and Methodology.

	Wave 1 (July 2013)	Wave 2 (April 2014)	Wave 3 (May 2015)
Location	Pingli County, Shaanxi Province	Shanyang County, Shaanxi Province	Danfeng County and Shangnan County, Shaanxi Province
Village Codes	1–3	4–10	11–17
No. of Caregivers Interviewed	10	10	40
No. of Grandmothers Interviewed	2	7	8

Our interview participants were primary caregivers of children in our target age range (6–18 months of age) living in our sample villages. In Table 2 we present the anemia rates in our sample villages. The WHO has estimated that about 20% of pre-school aged children (the youngest age group for which estimates were provided) in China are anemic [15], but rates of anemia are as high, if not higher, in all villages in our sample. We believe this makes our sample villages appropriate locations to study the determinants of complementary feeding practices. Children were randomly selected from a list of all registered births over the past six to eighteen months, which we obtained from the local health officials in each town/village. For each selected child, the primary caregiver was identified for inclusion within our qualitative sample; all were women. Because our sample was drawn from areas known to have high rates of child anemia, randomly selecting our sample within these areas allowed us to examine the extent to which opinions and beliefs pervasive on the community-level affect child feeding practices. Due to the minimal risk posed by our study procedures, written consent was not required by our ethical oversight committees.

**Table 2:** Anemia rate by village in rural Shaanxi Province, China.

Village code	Sample Size	Anemia rate (%)
Wave 1		
Village 1*	3	48.0
Village 2*	4	49.0
Village 3*	3	36.0
Wave 2		
Village 4	1	37.5
Village 5	2	66.7
Village 6	1	60.0
Village 7	2	20.0
Village 8	1	50.0
Village 9	1	33.3
Village 10	2	71.4
Wave 3		
Village 11	6	50.0
Village 12	8	44.4
Village 13	6	83.3
Village 14	8	25.0
Village 15	4	33.3
Village 16	3	25.0
Village 17	5	30.0

*Source*: authors’ own data

*In order not to bias the responses within our piloting testing phase, we did not collect Hb samples and conduct qualitative interviews within the same villages of Pingli County. The reported anemia rates are from villages neighboring those included in our qualitative sample, and we expect the anemia rates to be similar between these villages.

### Methodology

The three waves of qualitative interviews were conducted by fieldworkers associated with the Center for Experimental Economics in Education at Shaanxi Normal University and the West China School of Public Health at Sichuan University. Both institutions have experience in conducting qualitative fieldwork. Interviews were conducted by native Mandarin speakers as well as several foreign researchers fluent in Mandarin. At least one native Mandarin speaker was present at each interview. The interviews were semi-structured; interviewers referred to a scripted interview protocol, but also had the freedom to diverge from this protocol in order to investigate specific stories that emerged. Each interview lasted from thirty to ninety minutes. When permitted, the interviews were recorded. In cases where participants did not give permission for the interviews to be recorded (30 percent), all field members instead took detailed interview notes. Interview recordings were then transcribed and all personally identifiable information was removed from the transcripts.

Caregivers were asked a series of open-ended questions regarding their understanding of child nutrition, anemia, and malnutrition; their typical child feeding practices; their understanding of how to promote child development; and the sources of information that formed the basis of their understanding. The general topics covered within our interviews are listed in Table 3. Interview transcripts and field notes were subsequently translated into English for inclusion in the qualitative analysis. Each transcript was then anonymized; each caregiver was assigned a number which referred to the wave in which and the order in which they were interviewed. The qualitative analysis was conducted by members of the research team involved in the interviewing process in order to impose a degree of consistency on our research. Additionally, the interview responses from caregivers were supplemented with field notes and observations from the research team. The primary purpose of these field notes was to flag points of discussion and record information on the thoughts and impressions of the interviewers that could be returned to during the qualitative analysis.

**Table 3:** Topics of Questions asked in each phase of interviews.

Topics	Wave 1	Wave 2	Wave 3
Complementary feeding practices	x	x	x
Caregiver understanding on topics of child health and nutrition	x	x	x
Sources of information on nutrition	x	x	x
Cost constraints		x	x

*Source*: authors’ own data

Transcripts of interviews with caregivers were then coded for qualitative analysis. The coding process was conducted deductively using the themes listed in Table 3. Our qualitative data were then analyzed according to the following theoretical framework: Evidence suggests that high rates of anemia among children in rural China likely arise from micronutrient rather than caloric deficiencies [9, 10, 11]. In addition, due to previous work [9], we believe that factors exist that limit the ability of rural caregivers to adopt proper complementary feeding practices. The quotations presented in this paper were selected depending on how well they represented overall caregiver responses and if they offered new insights into reasons for current complementary feeding practices (or the absence of feeding practices that are normally accepted as standard/recommended).

## Results

Our interviews were conducted with 60 caregivers in rural Shaanxi province. Average characteristics of individuals from our sample areas are depicted in Table 4.

**Table 4:** Characteristics of individuals from our sample areas.

	Percent
Maternal age	
Age< = 25	48.5
Age>25	51.5
Maternal educational level	
< = 9 years	83.0
>9 years	17.0
Family receives Minimum Living Standard Guarantee	
No	76.1
Yes	23.9
Infant gender	
Male	48.0
Female	52.0
Infant age	
6–12 months	58.0
13–18 months	42.0
Mother is primary caregiver	
Yes	72.0
No	28.0

*Source*: authors’ own data

### Complementary Feeding Practices

Our qualitative interviews revealed that most caregivers do not follow the international recommendations for complementary feeding. Caregiver responses revealed that the diets of children consisted primarily of formula, starches, and fruit. Few (35.7%) children were fed vegetables daily and even fewer (23.2%) had meat in their diet (Table 5).

**Table 5:** Frequency of caregiver interview responses to feeding practices.

	Caregiver Responses (%)	
Type of Food	Does not feed to infant everyday	Feeds to infant one or more times a day
Breast milk	62.5	37.5
Formula	39.3	60.7
Starch	33.9	66.1
Vegetables	64.3	35.7
Meat	76.8	23.2
Fruit	32.1	67.8

*Source*: authors’ own data

The qualitative interviews revealed that most caregivers allowed their child’s preferences to dictate their diets, even to the point that children’s diets lacked foods that caregivers believed were healthy. If a baby refuses a food, caregivers often did not continue to attempt to feed it to the child. For this reason, several caregivers had stopped trying to feed their children vegetables. We found that caregivers continued to feed staple foods that the children would accept readily.

*“I just give her what she wants to eat*. *I don’t think different types of foods have different impacts on her development or growth*. *And I don’t make her eat what she doesn’t like*. *Even if I think something’s good for her*. *There’s no way to do it*. *If she doesn’t like something what can you do*?*” [2*.*5 Mother]*

*“If my baby doesn’t like the taste of the food*, *I won’t give it to her*. *She basically feeds herself*.*” [1*.*1 Mother]*

*“If he doesn’t like a food*, *he spits it out*. *Trying to feed it to him again is hopeless*. *So I don’t*.*” [1*.*9 Mother]*

*“The baby doesn’t like to eat vegetables so we don’t give them to him anymore*.*” [2*.*2 Grandma]*

*After her mom left the baby didn’t like the infant formula*. *So we didn’t give it to her*. *We just let her eat other things… In general*, *we just give her what she’s willing to eat*. *If she won’t eat something I give in and give her something else*.*” [2*.*3 Grandma]*

*“There are lots of foods he doesn’t like to eat*. *If he doesn’t like something I don’t give it to him*. *Like egg yolk is good for him*, *it’s nutritious*. *But he doesn’t like it so I don’t feed it to him*. *What can you do*? *He spits it out*.*” [2*.*8 Grandma]*

#### Timing of Introduction of Complementary Foods

We asked caregivers when complementary foods should be incorporated into a child’s diet. The most common responses from caregivers recommended breastfeeding and formula feeding exclusively until the child was one-year-old. Caregivers typically indicated that after a child’s first birthday starches and fruit should be introduced. Caregiver responses regarding when meat should be introduced into the diet varied from 15 to 24 months of age.

*“I started giving her meat when she was one and a half years old*. *Before that she wasn’t used to meat and would just get sick*.*” [1*.*1 Mother]*

*“From 0 to 10 months you should give the baby breast milk*. *After ten months you shouldn’t give any more breast milk*. *Then we switched to formula*. *And after five months you can start giving the baby a little bit of other solid foods*. *But we waited until about 8 months*. *Then we gave simple starches and a little bit of fruit*. *He doesn’t like vegetables so we don’t make him eat those*. *And we don’t give him any meat*. *I’m afraid that if we give him meat he will get diarrhea*. *I think we will wait until the baby is 2 years old to start giving him any meat*.*” [2*.*2 Grandma]*

*“Now the baby eats whatever the grownups eat*. *We don’t give her formula anymore*. *She’s almost two years old*. *We just give her some of what we are eating*. *Except for meat*. *We wouldn’t dare give such a young child any meat*.*” [2*.*3 Grandma]*

*“How would he eat vegetables*? *He’s too young*. *I’ll give him more vegetables when he is a little older” [3*.*03 Mother]*

*“I breastfed the baby for one year and then after she turned one I switched to formula*. *And I didn’t give her any other foods until she was a year and a half old*. *Now I feed her formula and noodles*, *porridge*, *tomatoes*, *and a little bit of meat sometimes*.*” [2*.*5 Mother]*

*“The baby has always been formula fed*. *He never had breast milk*. *We don’t give him many starches*. *Some porridge*. *He won’t eat other kinds of starches*. *We only started giving him porridge after he was one-year-old*. *And even now we have never given him fruit or veggies or meat*. *Mostly we just give him formula*. *About 150 mL three times per day*. *We will give him other kinds of food once he is willing to eat them*.*” [2*.*10 Grandma]*

*“The baby only had breast milk and formula until he was one-year-old*. *Then we started to give him some starchy foods and fruits and veggies*. *We didn’t dare give the baby any solid food before he was one year*. *Then it’s bad for him*. *Even if he had been willing to eat it at that age we wouldn’t have given it to him*. *And we waited a little longer to give meat*. *When the baby was about one year and three months we started giving him a little meat*.*” [2*.*1 Grandma]*

In addition to introducing complementary foods, most caregivers also reported changing their breast feeding habits at the one-year mark. Some caregivers expressed beliefs that after one year they could no longer produce enough breast milk to keep their baby full and that breast milk is less nutritious after one year.

*“I am planning to end breastfeeding when she is one-year-old*, *and then start feeding her formula*. *I am going to switch at one year because there is not enough breast milk at that time*. *Also*, *at one year my baby can adapt to drinking formula*.*” [1*.*10 Mother]*

*“At one year I will start giving him formula because that’s when the milk stops*. *It was the same with his sister*. *After that there is not enough breast milk*, *but formula is unlimited*. *Also*, *there aren’t enough nutrients left in the breast milk for it to be good at that time*.*” [1*.*5 Mother]*

#### Vitamins and Supplements

We found that most caregivers had little knowledge of vitamins and micronutrient supplements and many were unwilling to provide them to their child (62.5 percent). When asked if micronutrient supplements could improve physical and cognitive development, most caregivers answered in the negative. Many caregivers stated that they were not willing to feed products they are unfamiliar with to their children.

“We don’t dare give the baby anything to eat that we’re not familiar with. Even if people say it’s good for him.” [2.6 Mother]

*“We don’t give the baby any supplements*. *We don’t even give him the yingyangbao* [a micronutrient packet provided for free by the government in some of our study sites]. *The baby’s mother says it’s bad*. *They gave it to us to deceive us*. *So she doesn’t give it to him*.*” [2*.*9 Grandma]*

“The baby only needs vitamin supplements at two stages. First, when she’s learning to walk you have to give her supplements. Second, when she’s old enough to start school you should get her tested to see if she is deficient in any nutrients and maybe then give her some supplements. Apart from those two stages you shouldn’t give the baby any supplements.” [2.7 Mother]

As to vitamin supplements, caregivers often provided responses that supported the notion that vitamin supplementation was only necessary at certain developmental stages. Many caregivers cited delaying giving the child vitamins until they reached one year-of-age, stating that it was not healthy to give a baby supplements until they reached that age. Still, other caregivers believed that it was only the youngest children that needed vitamin supplementation and did not need to provide their child with vitamins past a particular age.

*“When the baby was less than one-year-old I gave him some vitamin supplements*, *but after that I stopped*. *After one-year-old the kid doesn’t need supplements*. *As soon as he can walk he doesn’t need them*. *Otherwise they can adversely impact his growth*.*” [2*.*7 Mother]*

### Caregiver Understanding on Topics of Child Health and Nutrition

Information gathered from our interviews demonstrated that caregivers’ often do not understand topics such as malnutrition and anemia, the relationship between nutrition and children’s growth and development, and the benefits of feeding children vitamins and supplements. Responses to interview questions on these topics are summarized in Table 6.

**Table 6:** Frequency of caregiver interview responses to questions reflecting level of knowledge on topics of nutrition.

		Caregiver Response (%)	
		Incorrect	Inadequate
Lack of understanding of anemia	The caregiver provided an incorrect or inadequate response to the question: *Do you know what anemia is?*	72.7	20.0
Lack of knowledge on the causes of anemia	The caregiver provided an incorrect or inadequate response to the question: *Can you explain the causes of anemia to me?*	72.7	21.8
Lack of knowledge on the consequences of anemia	The caregiver provided an incorrect or inadequate response to the question: *Can you explain the consequences to me?*	75.0	19.5
		No	
Lack of awareness of effect of diet on child development	Response of "no" to the question: *Do you think the type of food your baby eats has any effect on him/her?*	50.0	
Low rates of feeding children vitamins and supplements	Response of "no" to the question: *Do you feed your baby any vitamins or supplements?*	62.5	

*Source*: authors’ own data

#### Understanding of Malnutrition

Field interviews with caregivers revealed that there was little understanding of malnutrition amongst our sample. When asked to describe their understanding of malnutrition, many caregivers either lacked any comprehension of the term or responded to our questions in a manner that demonstrated they only had a limited or inaccurate understanding of the concept.

*“I don’t know what malnutrition is*.*” [1*.*5 Mother]*

*“Malnutrition is bad digestion*.*” [1*.*2 Mother]*

*“Malnutrition is when you don't absorb the food you’re eating so you’ll grow slowly*.*” [1*.*4 Mother]*

In certain interviews, we also asked caregivers if they knew what caused child malnutrition. Some caregivers appeared to venture guesses, but many of their assumptions and understandings were not correct.

*“It might be genes that cause malnutrition*.*” [1*.*7 Mother]]*

*“Malnutrition is when you get bad digestion from getting too many colds*. *It’s the result of catching colds easily*.*” [2*.*3 Grandma]*

*“Malnutrition is when you don’t have enough nutrition*. *But I’m not sure what impact malnutrition has on a person*. *The doctor said that the baby lacked calcium*, *but I don’t think that’s related to malnutrition*. *That’s something else*. *I’m not sure*.*” [2*.*1 Grandma]*

Further, while some caregivers demonstrated a general understanding that malnutrition can lead to disease, they could not identify of the actual causes and consequences of malnutrition. Caregivers cited visible physical signs as indicators of malnutrition. Some caregivers suggested remedies for malnutrition, but none of these included an improved diet that is higher in micronutrients.

*“You’ll know if your skin turns white and pale and you become really thirsty*. *Also*, *your hair would turn lighter*. *So dark skin is healthy…” [1*.*7 Mother]*

*“I don’t know what the effects of malnutrition are*. *But you can resolve it by curing the [common] cold*: *making the baby put on more clothes and sleep more*.*” [2*.*3 Grandma]*

*“If you have malnutrition*, *then you get diarrhea*. *If you get malnutrition you can cure it with medicine to promote better digestion*.*” [2*.*5 Mother]*

Some caregivers suggested remedies that would, in fact, be detrimental. We observed a belief common to many households that malnutrition is related to difficulty digesting foods. As a result, caregivers recommended eliminating or decreasing foods that are “difficult to digest,” which could potentially lead to removing micronutrient-rich foods from the child’s diet.

*“Malnutrition is when a child gets uncomfortable and bloated after they eat and can’t digest well*. *Maybe they only poop every several days*. *I’m not sure what causes malnutrition*. *You can cure it by feeding the baby less solid food*. *Give the baby more formula instead*. *You can’t let the baby eat so much*.*” [2*.*7 Mother]*

*“You can treat [malnutrition] by not eating things you can’t digest*.*” [1*.*2 Mother]*

*“Malnutrition is when you have trouble digesting certain foods like maybe noodles so you have to eat rice instead or something else that’s easier to digest*.*” [2*.*5 Mother]*

#### Understanding of Iron-deficiency Anemia

Similar to malnutrition, we found that our sample caregivers had a limited understanding of anemia. Many caregivers are unaware of the causes and symptoms of child anemia. When asked to identify the causes of anemia, many caregivers’ responses were loosely related to food consumption and nutrition; however, they did not demonstrate an understanding of the importance of micronutrients and a diverse complementary diet.

*“I’ve never heard of anemia*.*” [3*.*1 Grandma]*

*“Anemia*? *… Doesn't that mean you don’t have enough blood*?*” [1*.*2 Mother]*

*“I think you have anemia when you have low blood pressure and are a little dizzy*.*” [3*.*08 Mother*

*“[Anemia] is caused by not eating enough*.*” [1*.*9 Mother]*

*“Anemia is bad nutrition*.*” [2*.*6 Mother]*

Several caregivers appeared to venture guesses as to how to identify symptoms of anemia. In some cases, caregivers knew that it was necessary to treat anemia; however, they believed anemia to be prevalent only in adults and, therefore, thought that treatments are available only for adults. Many caregivers did not realize that it is possible for even a baby to have anemia. Others assumed that all children have anemia and will grow out of it as they age.

*“If you’re lightheaded and sweating a lot*, *you’re anemic*.*” [1*.*7 Mother]*

*“Before we found out [the baby] was anemic I’d never heard of anemia*. *Now I know that people with anemia have sallow*, *yellowish faces*.*” [2*.*4 Grandma]*

*“I’ve only heard of adults having anemia*, *not kids*.*” [1*.*6 Mother]*

*“My child has anemia*. *I’ve heard others say that all small children have anemia*. *When he is bigger it won’t be a problem*.*” [3*.*22 Mother]*

We asked caregivers to identify treatments for anemia and consequences that may arise if anemia is not treated in young children. We found that even when caregivers had an understanding of the term “anemia,” they often did not know how or did not think it was necessary to treat anemia. Other caregivers believed the only treatment for anemia to be medicine and did not recognize that a proper diet could prevent and treat anemia. Further, caregivers did not seem to be aware that the medicine prescribed was likely an iron supplement and therefore anemia could also be treated through complementary feeding.

*“I don’t know how to treat anemia*.*” [3*.*08 Mother]*

*“I’m pretty sure there’s no other way to cure anemia besides medicine*.*” [2*.*7 Mother]*

*“My grandmother has anemia*. *She’s been taking medicine*, *but I don't think they can cure it*. *I never thought of it as a big problem*. *I think people don't really care about anemia*.*” [1*.*4 Mother]*

*“My baby’s a little bit anemic*. *I know because some people came before and tested him for that*. *I’m anemic too*. *Sometimes when I stand up I feel light-headed*. *I’m not sure what causes it*. *I took some medicine for my anemia*. *But I didn’t worry about it when I found out the baby was anemic*. *He’s so young that we can’t tell if he’s uncomfortable or light-headed so we didn’t do anything for it*.*” [2*.*6 Mother]*

Not one mother referred to increasing iron in the diet through complementary feeding practices. Some caregivers did recommend increased consumption of formula, which could contain higher levels of iron. However, our interview respondents suggested feeding more formula *in lieu of* other foods–i.e., while decreasing complementary feeding.

*“We started giving the baby food other than breast milk when he was 11 months old*. *That’s when we started giving him formula instead and soft or liquid food like porridge*. *We didn’t give him any solid food before he was one-year-old*. *Otherwise it’s bad for him*. *We only give him a little meat because he can’t digest it very well yet*. *He gets constipated*.*” [2*.*9 Grandma]*

*“Mostly we just give him formula*. *About 150 mL three times per day*. *We will give him other kinds of food once he is willing to eat them*.*” [2*.*10 Grandma]*

*“You can cure [anemia] by feeding the baby less solid food*. *Give the baby more formula instead*. *You can’t let the baby eat so much*.*” [2*.*7 Mother]*

#### Understanding of Child Growth and Development

We asked caregivers if there is a connection between their child’s diet and his/her growth and development. Beyond a basic understanding of these terms, most rural caregivers simply do not believe there is a connection between diet and a child’s growth and development. We discovered that a common belief amongst caregivers that all food is nutritious, and, therefore, as long as a baby eats until he or she is full, the baby will get adequate nutrition.

*“It doesn’t matter what he eats*. *That doesn’t impact his development at all*.*” [2*.*6 Mother]*

*“Now I usually give him milk*, *I think whatever a child can eat is good for development*.*” [Mother 3*.*17]*

*“Different foods don’t affect the baby differently*. *All food has nutrition*. *As long as he eats his fill of course he will be healthy*.*” [2*.*8 Grandma]*

*“I don't think there’s a big impact of nutrition on health*. *Formula and porridge are good enough for my baby*.*” [1*.*2 Mother]*

Some caregivers acknowledged a connection between child nutrition and healthy development. However, these same caregivers suggested a diet consisting of starches, such as rice and porridge, as one that would stimulate physical and cognitive development. Interviews revealed a shared belief that dietary staples of formula and rice porridge provide adequate nutrition as long as the child consumes enough of it. Caregivers rarely mentioned the need to supplement this diet with other foods that are high in micronutrients, such as meats and vegetables. In fact, most caregivers believed that consuming such foods at this age actually affects development adversely and results in delayed growth and unhealthy outcomes, such as diarrhea.

*“Some foods have impacts [on my baby’s health]*. *Others don't*. *Soft*, *clean foods are good for my baby*. *Hard foods are not healthy*.*” [1*.*8 Mother]*

*“I don’t think about the effect of different kinds of foods on my baby*. *As long as she can eat her fill*, *I think that’s enough*. *If a certain food gives her diarrhea*, *then you shouldn’t give her that anymore*.*” [2*.*7 Mother]*

*“Yes*, *I have [thought that food choice will affect my baby’s cognitive development]… A good way to make him smarter is to feed him starches and rice*. *You can’t feed him hard food*. *You should feed him fewer vegetables*. *The effect of these foods is that he can grow taller*.*” [1*.*8 Mother]*

### Sources of Information on Nutrition

#### Family and Friends

We found that most caregivers rely on a combination of their own experience and the experience of friends and family members in making decisions regarding feeding their child. Additionally, we found that the sources of information caregivers relied on differed between mothers and grandmothers. Within our sample, 35 percent of mothers cited learning how to feed their child from their own experience, 31.7 percent learned from the experiences of friends, and 30 percent from the experiences of family.

*“I get tips for what to feed my baby from my mom and other family members*. *I have seen some stuff on TV and read some books*. *I trust everyone because most of it is consistent*, *or I just rely on personal experience*.*” [1*.*6 Mother]*

*“I get advice from friends and relatives*, *but I trust my own advice the most*. *I see how [my baby] is doing and decide whom I trust*.*” [1*.*8 Mother]*

*“I get information on taking care of my baby from other older people in the village*. *There’s no need to study*. *I know how to do it*. *Everyone says similar things*. *I do what I know how to do*.*” [2*.*5 Mother]*

*“I’m not really worried about the baby’s health*. *I am learning as I go*. *She’s my first child*. *If I ever don’t know what to do*, *I just ask my friends*.*” [2*.*7 Mother]*

We found that grandmothers frequently did not turn to any outside sources of information when it came to childcare and child nutrition. Many grandmothers claimed that because they had already raised children, they knew how to raise a healthy child, and therefore it was unnecessary to learn about new information concerning baby development.

*“We don’t need any nutrition or child care information*. *Everyone knows how to take care of babies*. *Just give the baby what she likes to eat*, *what she’s willing to eat*. *That’s all you have to do*.*” [2*.*3 Grandma]*

*“I raised my own children*. *I know what I’m doing*!*” [2*.*8 Grandma]*

Some caregivers did not know what to do when it came to caring for their children and described a feeling of futility about improving their knowledge. These caregivers turned to many different sources regarding child care and nutrition. However, they received inconsistent information from these sources, leaving them confused as to which sources of information were credible and which were not. We also found that some caregivers relied on television programs and advertisements to inform their nutrition choices.

*“I don’t think I have enough good information to take care of the baby*. *I don’t talk to other people about the baby*. *And I see ads about babies on TV sometimes but I don’t understand them*.*” [2*.*10 Grandma]*

*“I get lots of advice from my family*, *but they all say something different*. *I usually listen to whomever I disagree with least*.*” [1*.*2 Mother]*

*“I’m blindly buying the stuff companies tell us to buy*, *but I really don’t know anything or have any sources of information*. *No one ever told me how to feed my baby*. *I have no idea*. *I would read pamphlets in the hospital*. *Only then would I get information*. *But on an average day I don’t know anything*.*” [1*.*4 Mother]*

*“I can get good information on taking care of the baby from TV*.*” [2*.*9 Grandma]*

#### Health Professionals

Interviews revealed that many caregivers rarely interact with health professionals, such as doctors in their village or in the county seat. Therefore, these caregivers generally have few opportunities to solicit information on what to feed their infant. Additionally, those caregivers who do take their children to a village doctor reported either that they did not receive nutritional information, or that they did not trust the advice or information they were provided. As a result, many caregivers are often unwilling to rely on their village doctor as a source of information on how to care for their child.

*“This baby has never been to the doctor and has never had a checkup*. *The doctors around here [in our village] are no good*. *They can’t even treat the cold or diarrhea” [2*.*9 Grandma]*

*“He was found to be a little bit anemic… the doctor never said anything helpful to help me understand my child*. *He just told me to buy some rice powder and medicine*.*” [1*.*5 Mother]*

*“We’ve taken the baby to the village clinic before when he had a cold*. *But he’s never gone to the doctor or to get a health checkup*. *Doctors here don’t give nutrition advice; they just cure small problems*.*” [2*.*10 Grandma]*

While better information might be available from doctors in the county seat, many families live too far away for this to be a realistic option for obtaining health care or information.

*“I’ve never taken the baby to the doctor*. *For a routine check-up or anything else*. *We live very far away from the county seat*. *It’s not convenient to go there*.*” [2*.*5 Mother]*

*“We don’t usually take kids to the doctor here in the village*. *Instead we go to the county seat*. *But from here to the county seat it’s more than three hours*.*” [2*.*9 Grandma]*

#### Formula Vendors

Interviews revealed that in certain areas another source of information is the local formula vendor. Generally, it appeared that caregivers believed that the information provided by these formula vendors is reliable.

*“I read the booklets at the formula store for information*.*” [1*.*7 Mother]*

*“The person selling formula gives me tips about food*, *vitamins and supplements for my baby*. *“[1*.*3 Mother]*

*“His favorite thing to eat is a supplement*. *It’s basically a protein powder*. *It says on the box that the supplement is not suitable for babies under one year*, *but the formula vendor said it was okay*, *so I feed it to him anyway*.*” [1*.*4 Mother of a child that was less than one-year old]*

### Cost Constraints

In our interviews, caregivers consistently responded that cost is not a primary issue when it comes to providing for their children. Interview responses from caregivers in these households indicated that, although some families struggle to provide for their children, they nevertheless make sure to provide for their child’s nutritional needs to the best of their ability.

*“Money is not an obstacle for taking care of my baby*. *She’s still so young that she doesn’t have many needs*.*” [2*.*7 Mother]*

*“I buy what the baby likes to eat*. *Cost is not a major concern*.*” [2*.*1 Grandma]*

*“I can afford to take care of the baby*. *It’s not even a choice*. *If the baby needs something of course you have to find a way to get it for him*. *I would do anything for the baby*.*” [2*.*2 Grandma]*

*“Sometimes costs are a barrier to taking good care of my baby*. *We don’t get very much money from my husband’s income*. *But if the baby really needed something*, *if I thought she really needed something*, *I would get it for her*.*” [2*.*5 Mother]*

Additionally, conversations with caregivers suggested that families are capable of affording foods such as meats, fruits, and vegetables. However, although these foods are available, they are rarely provided to infants. Many caregivers indicated that they were unwilling, rather than unable, to provide their children with these foods.

*“And we waited a little longer to give meat*. *When the baby was about 1 year and three months we started giving him a little meat*.*” [2*.*1 Grandma]*

*“And we don’t give him any meat*. *I’m afraid that if we give him meat he will get diarrhea*. *I think we will wait until the baby is 2 years old to start giving him any meat*.*” [2*.*2 Grandma]*

*“Now the baby eats whatever the grownups eat*. *We don’t give her formula anymore*. *She’s almost two years old*. *We just give her some of what we are eating*. *Except for meat*. *We wouldn’t dare give such a young child any meat*.*” [2*.*3 Grandma]*

## Discussion

Field interviews with mothers and grandmothers provided insight into the causes of complementary feeding practices that may contribute to the high incidence of malnutrition and anemia among children in rural Shaanxi Province, China. In all, our qualitative interviews suggest that few caregivers in rural Shaanxi province practice age-appropriate complementary feeding. From our interviews, we discovered that caregivers rarely incorporate solid foods into a child’s diet until they are at least one year-of-age, and once solid foods were incorporated they rarely included micronutrient-rich meats and vegetables. Complementary feeding practices are inconsistent with those recommended by the WHO, which suggests that caregivers begin to incorporate solid foods into an infant’s diet starting when they are around six months-of-age and feed their child some form of meat, poultry, fish, or egg and fruits and vegetables daily [2]. Instead, our sample caregivers systematically are waiting until children are older to begin to incorporate solid foods, and when they do they primarily provide starches and only small quantities of fruit. Additionally, caregivers often cease to provide their child with breastmilk or formula as they begin to introduce solid foods. The deficit of iron-rich foods, such as meat and vegetables, from this diet may contribute to the high prevalence of iron-deficiency anemia among infants in our sample villages.

From our interviews we have also gained understanding on reasons *why* rural caregivers engage in these complementary feeding practices. It appears that complementary feeding practices are constrained by a lack of understanding of infant nutrition rather than the cost of providing a nutritious diet to children. Although Luo et al. (2014) found that nearly one-fourth of the families in our sample areas received Social Security Support (payments from China’s Minimum Living Standard Guarantee system) [9], caregivers included in our qualitative sample consistently stated that financial constraints were not an issue when attempting to provide for their children. The interview questions related to costs repeatedly drew responses from the caregivers indicating that their complementary feeding practices and decisions are not resulting from their poverty status. This finding is supported by Luo et al. (2014), who found high rates of anemia accompanied by low rates of child wasting and stunting, a pattern that can occur when children have sufficient caloric (and other macronutrient) intake, but insufficient micronutrient intake [9], suggesting that it is a deficient quality, not quantity, of food that may be leading to child malnutrition in rural China. Additionally, caregivers provided answers that lead us to believe that nutritious food is relatively affordable, but not provided to infants. Thus, we sought to determine what other factors are in place that constrain adequate feeding practices.

We believe that inadequate complementary feeding practices arise from an absence of understanding on topics of infant health and nutrition. Many caregivers shared the belief that anything a child eats will provide him or her with adequate nutrition and that it is the quantity of food, rather than the quality, that leads to malnutrition or anemia amongst infants. Due to this belief, we also found that caregivers are not likely to feed their child foods they do not like, even if the caregiver recognizes that the food is healthy. Caregivers cited ceasing to provide breast milk to their children around one year-of-age under the reasoning that the milk was not as nutritious after one year, and would instead switch to formula feeding in some cases (or a completely solid food diet in other cases). Caregivers were also hesitant to provide their infants with vitamins or supplements due to the belief that such products should not be given to such young children.

Misconceptions concerning complementary feeding practices were also coupled with a lack of understanding on topics of child health and nutrition. Our qualitative interviews revealed that few caregivers had any understanding of malnutrition, anemia, or the relationship between an infant’s diet and their development. Many caregivers recognized that malnutrition could lead to disease, generally, but could not name causes or specific consequences of malnutrition. Of concern, we found that some caregivers believed malnutrition was related to eating hard-to-digest foods, and therefore would cut micronutrient-rich foods (primarily meat) out of a child’s diet. Caregiver knowledge of anemia was similarly deficient. Few caregivers were capable of describing the causes, manifestations, and consequences of anemia. Even those caregivers who had a basic understanding of anemia believed that it can only be cured through medicine and did not cite the importance of a micronutrient-rich diet. The results are troubling, as they suggest caregivers do not recognize the threat infant anemia places on their child’s development and do not know how to prevent iron-deficiency or treat it when it arises.

We believe that a primary cause of misconceptions concerning complementary feeding practices, malnutrition, and anemia arise from an absence of sources of information on topics of child health and nutrition. Our interviews revealed that the majority of caregivers rely on their own experiences or the experiences of others (who may be just as misinformed, such as their mother-in-laws or their friends from neighboring villages) to inform their feeding practices. In certain cases, this reliance on the child-rearing experience of others can be detrimental to child development, since other caregivers in the community likely do not have access to any better sources of information. Therefore, this system of shared knowledge perpetuates the community’s unawareness of sound child feeding practices. In several interviews, we also found that some caregivers listen to the nutritional advice of formula salesmen, who we might expect to have primarily profit-seeking motives that might bias their advice.

Even if caregivers were eager to improve their knowledge on child nutrition, they do not have access to quality sources of information. Caregivers rarely interact with doctors or other medical professionals, and many do not trust the information provided. Caregivers cited receiving misleading advice from rural doctors, often resulting in the belief that village doctors were incapable of providing appropriate nutritional information. A recent study conducted in rural Shaanxi Province on the quality of village doctors supports this belief. This study used standardized patients who were trained to simulate a set of symptoms to measure quality of care. The results showed that village clinicians provided unnecessary or harmful medications to patients 64% of the time [16]. Therefore, it is not surprising to find a distrust of rural physicians among rural caregivers.

Another potential reason for these low levels of positive infant nutritional outcomes may be the low rate of formal educational attainment in rural China. Only 17 percent of mothers within our sample areas received more than nine years of schooling. Previous research has demonstrated that poor health outcomes are significantly correlated with low levels of maternal formal education [9] and that increased maternal formal education has a positive effect on child health outcomes [17–19]. Within the context of rural China, an even bigger concern is that grandparents are likely to have received even less formal education than mothers and, therefore, know even less about health and nutrition [20]. However, this is not to say that low levels of formal education among caregiver prohibit the adoption of correct health and nutritional practices. Research has found that although maternal knowledge on nutrition is a positive function of maternal formal education, it is maternal knowledge on nutrition, and not formal education, that positively affects child micronutrient status [21]. For this reason, it may be possible to improve health and nutritional practices among caregivers in rural China by providing nutritional education and increasing their knowledge on these subjects.

### Implications for Public Health and Policy

The persistence of micronutrient deficient diets among young children in rural China is of particular concern due to the lasting impacts of nutritional status at this age on growth and life-long cognitive and physical development. This importance is demonstrated by the research of Chen et al. (2010), which employed a multi-arm quasi-experimental study among children aged 4–12 months of age in rural Gansu Province. This study found that significant differences in physical and cognitive development not only emerge between children who do and do not consume sufficient calories, but also between those who are and are not supplied a micronutrient-rich diet, whether or not energy intake is sufficient. Additionally, the effects on children’s intellectual development were found to persist and gradually increase at least until 6 years of age [14]. These findings are important, as they not only demonstrate that complementary feeding practices among rural Chinese caregivers are insufficient, but also depict the long-term effects of encouraging complementary feeding practices and increased micronutrient consumption at a young age. Therefore, improving the feeding practices of caregivers could potentially improve human capital development across China’s rural population.

One method that holds promise for improving infant feeding practices is that of local public health and nutrition campaigns, as recent research has demonstrated such campaigns may improve the nutritional outcomes of infants and young children in rural China. Shi et al. (2009) demonstrated the effectiveness of health campaigns covering topics of feeding practices and child nutrition among caregivers of young children in rural Hebei Province. This study implemented an educational intervention focusing on improving complementary feeding practices among caregivers of children aged 2–4 months at baseline, and found that the food diversity, meal frequency, and hygiene practices of intervention caregivers improved within the study period, suggesting that health campaigns can change caregiver behavior [22]. Additionally, due to findings that the first 18 months of life are a critical time for the prevention and correction of anemia [14], encouraging proper complementary feeding practices among young children may be the most effective way to prevent anemia across an individual’s lifespan.

In order to improve complementary feeding practices among rural caregivers, health and nutrition campaigns would be well-served to focus on topics such as appropriate timing for introducing new foods and how to provide children with a micronutrient-rich diet. In addition to providing education to caregivers on topics of complementary feeding, educational campaigns of this nature that focus on micronutrient supplementation may be particularly useful, as recent interventions have been successful in reducing child anemia in rural China [23]; however, more research is warranted to identify the most effective means of large-scale implementation. Due to the general lack of trust of local physicians found in our qualitative interviews, it is likely that caregivers would need these educational materials to come from other, more trusted sources.

We recommend that local government workers who have relatively easy access to villages and who (in recent years) have established more positive relationships with villagers be trained in healthy child nutrition and complementary feeding practices. The most obvious partner in rural China today would be the local (town-level) division of the Population & Family Planning Commission (PFPC). Because historically, the PFPC has been responsible for the enforcement of China’s one-child policy, there is a small set of officials based in each township that has relatively easy access to every village in China. With the end of China’s one-child policy, the PFPC is looking for a new institutional mission, and has turned its attention to early child development [24]. Given that the PFPC already has the institutional reach, the bureaucratic capability and considerable experience conducting village outreach and running informational campaigns, it seems to be a feasible possible solution to be used as a resource for disbursing information on child nutrition. Addressing the nutritional literacy of caregivers holds promise as a method to improve the overall health and development of millions of rural children, and therefore deserves consideration in the formulation of future public policy.

### Study Limitations

The study sample was comprised of sixty households in seventeen rural villages across four counties in Shaanxi Province. We attempted to sample villages that varied in terms of household income, population size, distance from the county seat, and geographic location. However, it is possible that the sample is not representative of all households throughout rural Shaanxi Province (or all of rural China).

Caregivers in our sample were selected from villages that were included in a larger survey of child health and nutrition outcomes consisting of 1808 families. While we have information on the prevalence of child anemia among the larger study population, we do not have this information for the children in our smaller qualitative sample. Therefore, we are only able to speak to the prevalence of poor complementary feeding practices within this paper and cannot link these practices to the high rates of anemia within our sample areas.

We identified participants for the study based on a list of registered children provided by the village family planning official. As a result, all unregistered children were systematically excluded from the sample and may be a potential source of bias. Although there is evidence that this issue (children being unregistered) is no longer as pervasive as it was in the past [25], it is possible that we did miss this segment of the population. Additional bias stems from the reality of conducting qualitative interviews, though we attempted to control for this by maintaining consistency in the composition of our research team throughout the course of the study.

## Conclusion

Our results suggest shortcomings in the understanding of basic knowledge on infant weaning and on overall health and nutrition among rural caregivers, this may lead to poor complementary feeding practices that in turn may contribute to the high prevalence of malnutrition and iron-deficiency anemia among rural infants in China. Though poor complementary feeding is prevalent in poor areas of rural China, our qualitative findings suggest that poverty itself does not constrain feeding practices and caregivers attempt to provide for their children as best as they can. Therefore, the absence of nutritional knowledge needs to be addressed by future policies in order to improve long-run health and human capital outcomes in China.

In order to combat the high prevalence of inadequate feeding practices, we suggest implementing nutritional campaigns targeted at caregivers of young children in rural China that focus primarily on promoting micronutrient supplementation. Such an educational campaign can be carried out by local government workers who have established positive relationships with villages and therefore would be a trusted source that caregivers could turn to when seeking nutritional information. Implementing such a measure can both increase caregivers' access to reliable sources of information, and their knowledge of infant and child nutrition. By doing so, these measures may effectively combat some of the most serious impediments to proper children's feeding practices and consequently their nutritional status, health, growth and development in rural China.
